# Image-Based Automatic Watermeter Reading under Challenging Environments

**DOI:** 10.3390/s21020434

**Published:** 2021-01-09

**Authors:** Qingqi Hong, Yiwei Ding, Jinpeng Lin, Meihong Wang, Qingyang Wei, Xianwei Wang, Ming Zeng

**Affiliations:** 1 School of Informatics, Xiamen University, Xiamen 361000, China; hongqq@xmu.edu.cn (Q.H.); dingyiwei@stu.xmu.edu.cn (Y.D.); jplin@stu.xmu.edu.cn (J.L.); wangmh@xmu.edu.cn (M.W.); 24320182203282@stu.xmu.edu.cn (X.W.); 2 School of Automation and Electrical Engineering, University of Science and Technology Beijing, Beijing 100083, China; weiqy@ustb.edu.cn

**Keywords:** watermeter reading, automatic method, neural network, deep learning

## Abstract

With the rapid development of artificial intelligence and fifth-generation mobile network technologies, automatic instrument reading has become an increasingly important topic for intelligent sensors in smart cities. We propose a full pipeline to automatically read watermeters based on a single image, using deep learning methods to provide new technical support for an intelligent water meter reading. To handle the various challenging environments where watermeters reside, our pipeline disentangled the task into individual subtasks based on the structures of typical watermeters. These subtasks include component localization, orientation alignment, spatial layout guidance reading, and regression-based pointer reading. The devised algorithms for orientation alignment and spatial layout guidance are tailored to improve the robustness of our neural network. We also collect images of watermeters in real scenes and build a dataset for training and evaluation. Experimental results demonstrate the effectiveness of the proposed method even under challenging environments with varying lighting, occlusions, and different orientations. Thanks to the lightweight algorithms adopted in our pipeline, the system can be easily deployed and fully automated.

## 1. Introduction

Automation is widely used to optimize processes and facilitate labor-intensive tasks in our daily life. In the field of smart cities, with the development of technologies of artificial intelligence and fifth-generation mobile networks, as a core part of the intelligent sensors, the technique of automatic device reading has become increasingly critical and technically feasible. Automatic watermeter reading is one such practical and challenging task. In recent years, many methods related to automated meter reading have been proposed to make this work more convenient.

Current automatic watermeter reading methods, however, do not generalize well to typical daily usage scenarios. One kind of current method involves equipping a miniature camera to the watermeter [1,2,3,4]. Although effective, this increases expenses because of the additional camera setup required for each watermeter. Another kind of method is sensor-based. These methods [5,6,7] take an alternative approach, and integrate wireless transceivers into the watermeter. Water flow can be sensed by performing adaptive signal processing on the generated voltage to provide real-time water flow information.

**Figure 1:**
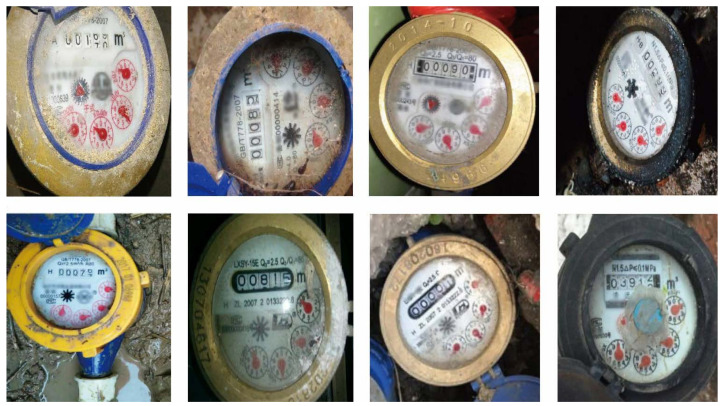
Watermeters under different challenging environments in real life.

Both types of smart readers methods described above require high switching costs. Despite the existence of smart readers, they are not widespread in many countries, especially in the underdeveloped ones, and it is still manually read on site by the operator who uses the image as proof of reading. Moreover, since there are many images to be evaluated, the traditional manual reading of the watermeter is tedious and error-prone. To address this dilemma, we propose taking a photo with a mobile phone and automatically analyzing the watermeter reading in this image. Because of the diversity of imaging conditions, especially the potentially challenging environments where the watermeters reside, it is not a trivial work to develop a robust automatic reading system for watermeters. As shown in Figure 1, real images of watermeters come with a variety of challenges. First, the lighting, resolution, and background environment varies across images. Second, the position and rotation angle of the watermeter is unpredictable. Finally, the watermeter is usually covered with dust, and the resolution of the image is low, making it tough to accurately read the watermeter value.

To solve these challenges, we leverage the techniques of CNN-based deep learning [8], including object detection [9], classification [10], and regression [11,12], to complete watermeter reading automatically. Our method requires manual taking the watermeter image and can automatically take this reading, which avoids the tedious reading process and ensures the reading’s accuracy in a low-cost way, effectively preventing artificial tampering with data and significantly improving efficiency and accuracy.

This paper explores object detection, orientation alignment, spatial layout guidance digit localization, and value regression to implement automatic watermeter reading. We use object detection to extract the position of the watermeter in the input image and crop it out. Then, the orientation alignment algorithm is utilized to adjust the cropped image to the correct reading orientation. Next, we extract the part of the watermeter that contains the reading information (a digit box and several pointer meters). Finally, we utilize the spatial layout guidance algorithm to locate each digit and then read the digits and pointer values to obtain the final reading. Particularly under challenging environments, the devised orientation alignment ensures that the reading task is executed at the correct angle; additionally, the spatial layout guidance algorithm helps us locate each digit accurately. Based on these building blocks, we propose an end-to-end pipeline and train it on a large and challenging environment dataset, yielding a robust automatic reading system for watermeters.

In summary, the contributions of this paper are as follows:

- We propose a robust end-to-end system based on convolutional neural networks for automatic reading of structured watermeter instruments. Our method tailors and combines the latest object detection, feature point location, and novel angle regression techniques.
- We design an orientation alignment algorithm for image correction and propose a spatial layout guidance algorithm to locate digits.
- We carry out a comprehensive experimental analysis that shows that our method effectively meets the challenges of various environmental factors and achieve reliable meter reading performance.
- We build a large-scale watermeter dataset including 9500 training images and 500 test images. To the best of our knowledge, this is the largest watermeter dataset with images taken under different challenging environments. This dataset can further improve the robustness of our automatic readings.

## 2. Related Work

This section reviews the methods employed for the automatic reading of instruments and introduces the procedures we utilize for automated meter reading, object detection, and text detection.

### 2.1. Automatic Meter Reading

There are many types of automatic meter reading usage scenarios in real life. The most widely used are pointer reading and digit reading. In terms of pointer reading, the traditional computer vision method combines the binary image subtraction [13] and the Hough transform [14] to estimate the angle of the pointer. However, this method is not robust enough for complex environments, such as various backgrounds and lighting. Zuo et al. [15] improved the existing Mask-RCNN approach [16], classifying the type of pointer meters while predicting the pointer binary mask and then calculating the readings of a pointer table according to the angle of the pointer. However, this method is designed for a specific environment, which reduces the method’s application scope in a real-world environment. As for digit reading, Anis et al. [17] propose recognizing digital meter reading based on the Horizontal and Vertical Binary (HVB) patterns. But the digit numbers they process are complete and static, which is not suitable for rolling digit meter reading in a watermeter. Laroca et al. [18] employs the Fast-YOLO object detector for components detection and evaluates three different CNN-based approaches for components recognition. They regarded the reading of the rolling digit as the goal of future work and did not propose a solution. Many researchers have concentrated on improving digit recognition algorithms [19] or classifiers [20] to achieve higher precision in digit recognition. Although previous mentioned digit recognition methods [21,22] have reached very high accuracy, they are not suitable for the watermeter reading task because the digit will roll with the volume of water. For example, for a value of 0.5, the digit turns between 0 and 1 and does not concentrate on 0 or 1. We use the probability distribution matching to solve this problem, the details of which will be introduced in Section 3.4.

### 2.2. Object Detection

To read a meter, we must locate the digits that need to be read. We use an object detector to predict the positions of the digits that we need to read. Some recent approaches exploited the vertical and horizontal pixel projections histograms [23] for object detection. Other methods took advantage of prior knowledge, such as object position or its colors [24]. These techniques’ inevitable shortcoming is that they might not work on all meter types, and the color information might not be stable when the illumination changes. Therefore, we utilize a Convolution Neural Network (CNN)-based object detector to locate the water meter and its inner components. Common CNN-based object detection algorithms can be divided into two categories: two-stage detection algorithms, and one-stage detection algorithms. The former divides the detection problem into two stages. The first stage generates candidate regions, and the second stage classifies candidate regions. The most representative two-stage object detector is the R-CNN [25] series, including fast R-CNN [26], faster R-CNN [27], R-FCN [28], and Libra R-CNN [29]. Meanwhile, one-stage detection algorithms include SSD [30,31] and YOLO [32,33,34], and do not require the region proposal stage; instead, these algorithms directly generate the category probability and position coordinate value of the object. The two-stage algorithm is accurate and the one-stage algorithm is lightweight. After several version updates, YOLO3 [34] not only reached higher accuracy but also maintained a high running speed. Therefore, we leverage YOLO3 as our watermeter detection model.

### 2.3. Text Detection

The major trend in scene text detection before the emergence of deep learning was bottom-up, in which case handcrafted features were used most often, such as SWT [35] or MSER [36] as a basic component, but those algorithm failed with bluring and perspective distortions. The current widely used text detectors are as follows: the regression-based text detectors [37,38] adopt object detection methods to find the position of words; the segmentation-based text detectors [39,40] aim to find the pixel-level text area and detect the text by estimating the word boundary area; and character-level text detectors [41,42] detect the text area by exploring each character and the affinity between characters. A major drawback of these techniques is that their results are susceptible to non-text lines.

## 3. Proposed Method

### 3.1. Reading Rule of Mechanical Watermeters

**Figure 2:**
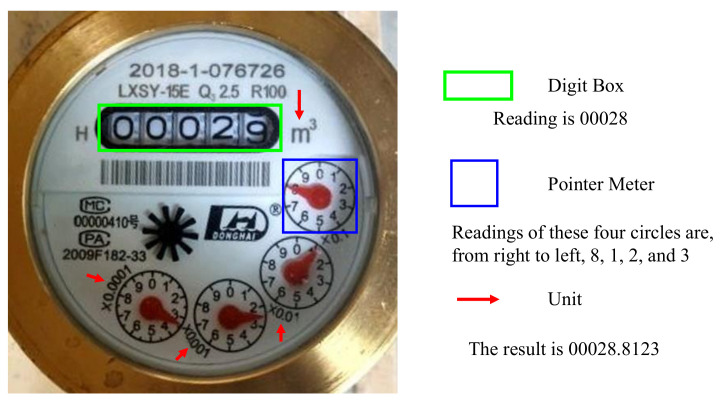
Schematic diagram of the watermeter. The weighted sum of the values of the digit box and the pointer meter indicates the water consumption.

Figure 2 depicts a typical mechanical watermeter. It is composed of structured digit panels and corresponding units. Although the structures of watermeters produced by different companies are not the same, they share similar panel layouts and reading rules. Therefore, an automatic reading watermeter method based on the divide-and-parse methodology can be easily adapted to various watermeters with similar structures. Figure 2 also illustrates the reading rule for a typical watermeter. The green box contains the digit box’s value (reading this is called digit reading), and the blue box contains the pointer’s value (reading this is called pointer reading). The red arrows point to the corresponding units. A weighted sum of these values results in the final water usage reading.

### 3.2. Overview

**Figure 3:**
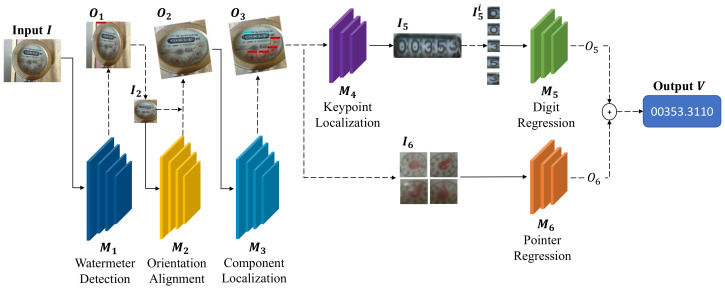
The pipeline of our framework. Our model takes a watermeter image *I* as input and outputs the corresponding value *V*. First, the detection model $M_{1}$ detects the position $O_{1}$ of the watermeter, resulting in the cropped watermter image $I_{2}$. Second, the orientation alignment module $M_{2}$ is adopted to get the aligned image patch $O_{2}$. Then, the component localization module $M_{3}$ predicts and locates the digit box $I_{5}$ and the regions of pointer meters $I_{6}$. Next, the keypoint localization model $M_{4}$ is introduced to localize and separate each digit in $I_{5}$. Finally, the values $O_{5}$ and $O_{6}$ are obtained by the digit reading model $M_{5}$ and the pointer reading model $M_{6}$, respectively. The final prediction *V* is the sum of $O_{5}$ and $O_{6}$.

According to the reading rule of the mechanical watermeters, and considering the potentially challenging environments in which watermeters are located, we split the reading task into individual subtasks. Figure 3 shows the full pipeline of our system framework. The whole pipeline consists of the following parts: watermeter detection, orientation alignment, digit reading with spatial layout guidance and pointer reading.

Our pipeline takes a watermeter image *I* as input and then it outputs the corresponding watermeter value. First, the watermeter detection model $M_{1}$ detects the position $O_{1}$ of the watermeter and then obtain the cropped watermeter image $I_{2}$. Second, we adopt the orientation alignment module $M_{2}$ to rotate $I_{2}$; this is followed by component localization with component detection module $M_{3}$, leading to one bounding box $I_{5}$ for the digit box and the four bounding boxes $I_{6}$ for pointer meters. Then, we design the keypoint localization model $M_{4}$ to localize and separate each digit in $I_{5}$. Finally, we can read the values $O_{5}$ and $O_{6}$ by the digit reading model $M_{5}$ and the pointer reading model $M_{6}$, respectively. The final prediction *V* is the sum of $O_{5}$ and $O_{6}$.

### 3.3. Watermeter Detection and Rotation Corrected Component Localization

#### 3.3.1. Watermeter Detection

To accurately and rapidly determine the position of the watermeter, we adopt YOLO3 [34] for one-class (only the watermeter) object detection. Taking an image *I* as input, YOLO3 output the position ${\left(x,y,w,h\right)}_{m}$ of the watermeter. The position information includes the center ${\left(x,y\right)}_{m}$, the width (*w*), and the height (*h*), where *m* represents the subscript of detection results.

#### 3.3.2. Orientation Alignment

**Figure 4:**
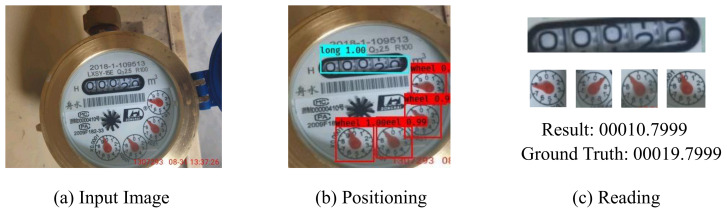
Failure case of incorrect reading caused by the inaccurate localization for the digit box.

Because of the 6-DOF transformation of the camera viewpoint, the cropped region $I_{2}$ has a perspective transformation. As a result, direct positioning and reading caused problems: as shown in Figure 4, the positioning of the number box is offset (Figure 4b) and the reading is incorrect (Figure 4c).

Therefore, we propose an orientation alignment network to adjust the reading angle. Our method predicts an angle of in-plane rotation to correct the orientation of $I_{2}$. Although the transformation is actually perspective, in practice the simplification of in-plane rotation is sufficient to account for this varaiation and achieve satisfactory results. More concretely, we did not directly regress the rotation angle because the angle is periodic. For example, −20° and 340° correspond to the same angle, which is ambiguous. To eliminate this ambiguity, we regress the sin and cos values of a given angle. Hence, the loss function can be formulated as follows:

(1) $$L_{angle}={∥P_{sin}-sin\left(\theta \right)∥}^{2}+{∥P_{cos}-cos\left(\theta \right)∥}^{2},$$

where $P_{sin}$ and $P_{cos}$ is the output of $M_{2}$. θ∈[−π,π] denotes the ground truth angle.

#### 3.3.3. Component Localization

We use another YOLO3 detection model $M_{3}$ for two-classes (the digit box and the pointer meters) object detection. Using the image $O_{2}$ as the input, $M_{3}$ output the position of the digit box and pointer meters, denotes as ${\left(x,y,w,h\right)}_{d}$ and ${\left\{{\left(x,y,w,h\right)}_{p}^{j}\right\}}_{j=1}^{4}$.

### 3.4. Regression-Based Digit Reading with Spatial Layout Guidance

#### 3.4.1. Spatial Layout Guidance for Digit Localization

Because of the low image quality caused by bad environments, straightforward methods like uniform character segmentation and Optical Character Recognition (OCR) text detection may fail to locate and read each digit accurately. We have explored two attempts:

1. Given the detected digital region, we uniformly separate each digit and then predict the value for each digit using regression.
2. Directly leverage an off-the-shelf OCR module to recognize the digits.

**Figure 5:**
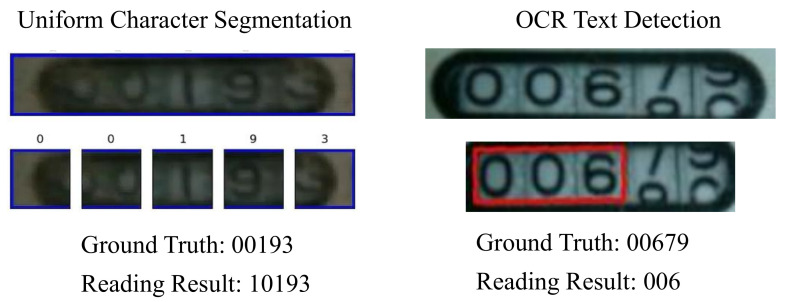
Uniform character segmentation and character detection. The segmentation result on the left is inaccurate, and the digits on the right are not fully detected; both lead to wrong readings.

However, neither of these methods accurately locates the position of each digit. The disadvantages of these straightforward methods are illustrated in Figure 5. The first method relied too heavily on the results of the component localization model $M_{3}$. If the results of the $M_{3}$ are not accurate enough, incomplete digits will be generated. Meanwhile, the second method often fails when recognizing a rolling digit, which is a common occurrence in watermeters.

**Figure 6:**
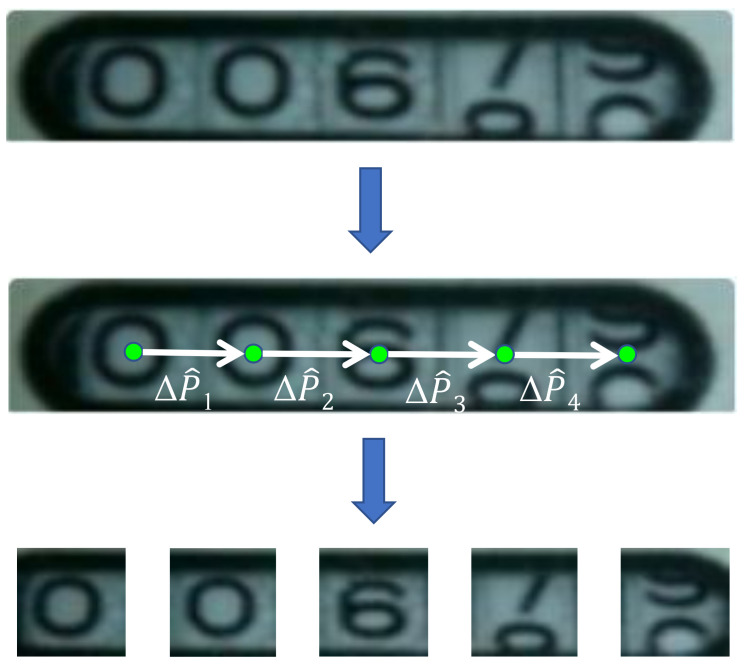
Keypoint localization method with spatial layout guidance. The five green points are on the same line and are equidistant.

To address the problem of digital localization, we recast the problem as keypoint localization. We locate the positions of digits $\left(x_{i},y_{i}\right)$i=(1,2,…,5). Because of the low image quality and the transition state between two consecutive digits, the text is not clear enough to robustly localize the digits. We thus utilize the linear spatial layout of the text region as prior information to constrain the keypoint localization. Therefore, we require that the predicted localization of each digit is colinear and equidistant (as illustrated in Figure 6), which could be formulated as neighboring offsets of predicted positions are almost the same. Hence, the loss function is as follows:

(2) $$L_{keypoint}=\sum_{i=1}^{N}{∥P_{i}-{\hat{P}}_{i}∥}^{2}+\sum_{i=1}^{N-1}{∥\Delta {\hat{P}}_{i}-\Delta {\hat{P}}_{i-1}∥}^{2},$$

where $P_{i}$ denotes the ground truth of the 2D coordinate of the ith digit position and $\hat{P}$ denotes the predicted coordinate, and $\Delta {\hat{P}}_{i}$ = ${\hat{P}}_{i+1}-{\hat{P}}_{i}$ denotes the spatial offset of nearby digits.

#### 3.4.2. Digit Reading

**Figure 7:**
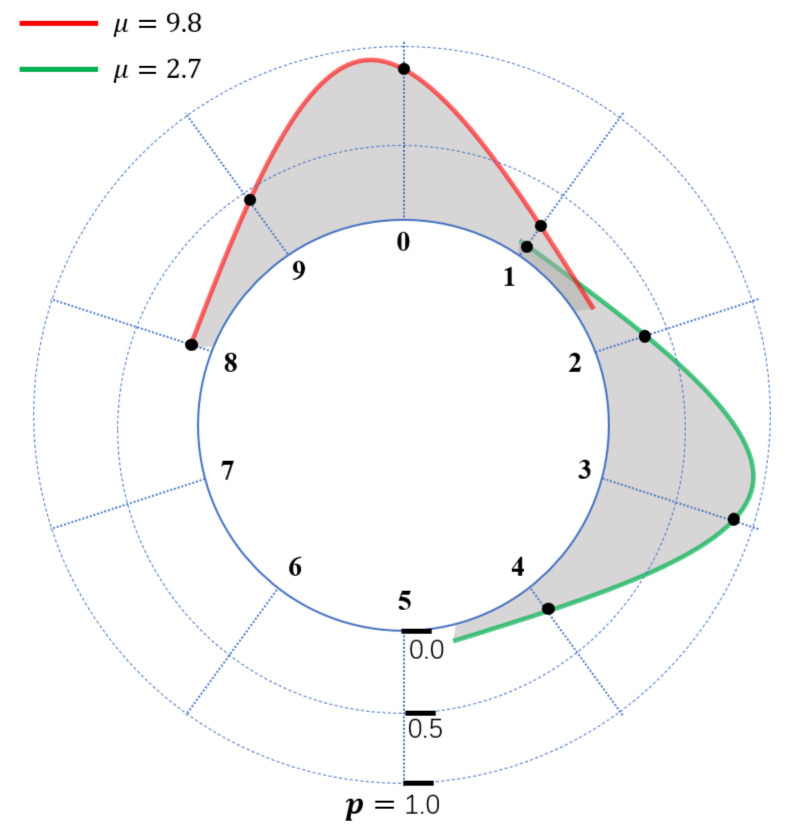
Gaussian Circle Probability Distribution (CPD) centered at different μ values. For each discrete integer in the range [0,9], we sample the corresponding probability(the probability at the black dot) to construct the final ground truth.

After keypoint localization, we crop the digit box images into five parts according to regressed coordinates. For the ground truth of 9.5, as shown in the far-right digit in Figure 6, the digit appeared as the bottom half of 9 and the top half of 0. A straightforward way to predict the digit value is to regress the value between [0,10) by using Mean Square Error(MSE). But the penalty is different when the model output 0.0 and 9.0 with MSE loss (${\left(9.5-0\right)}^{2}\;\mathrm{vs}.\;{\left(9.5-9.0\right)}^{2}$), which will provide the wrong update information to the model. This situation is caused by the value jump from 9 to 0. To eliminate this effect, we formulate this task as a Circle Probability Distribution (CPD) prediction problem. Specifically, as shown in Figure 7, we use a Gaussian distribution $N(\mu ,\sigma^{2})$ to calculate probabilities for every discrete integer (ranging from 0 to 9 by step size of 1). Given μ as the ground truth, we set σ=0.05 in our experiment, and the optimal model should predict a Gaussian CPD centered at μ.

We use a CNN module denoted as $M_{5}$ for the digit reading. Given input images $I_{5}^{i}$, we can sample ten probabilities ${\left\{p_{i}\right\}}_{i=0}^{9}$ as noted earlier, and then $M_{5}$ output ten probabilities ${\left\{{\hat{p}}_{i}\right\}}_{i=0}^{9}$. Categorical cross-entropy loss is introduced to fit the Gaussian CPD, as follows:

(3) $$L_{digit}=-\frac{1}{N}\sum_{i=1}^{N}\sum_{j=0}^{9}p_{i}^{j}log{\hat{p}}_{i}^{j},$$

where *N* denotes the training sample number. We assume that the maximum probability(the crest of CPD) locates between indices of $I_{1}$ and $I_{2}$ with the top-2 predicted probabilities in $\left\{{\hat{P}}_{0},{\hat{P}}_{1},\ldots ,{\hat{P}}_{9}\right\}$. We use min($I_{1}$, $I_{2}$) as the final output $O_{5}$ according to the watermeter reading rule.

### 3.5. Regression-Based Pointer Reading

To read the value of pointer meters, we first crop $O_{2}$ using ${\left(x,y,w,h\right)}_{p}$ and obtain image $I_{6}$. The rotation angle of the pointer could infer the pointer’s value. Because the pointer in the dial meter is discriminative, it is a more natural problem for the neural network to learn the direction of the pointer. To estimate the angle of the pointer, as in Section 3.3, we trained the orientation alignment model to regress the cos and sin values of the pointer angle. We use the same regression loss to train the pointer reading model.

## 4. Experiments

In this section, we first describe the data preparation and the evaluation metrics of our experiments. Then we verify the effectiveness of our proposed method from three aspects: (1) We conduct quantitative and qualitative experiments to demonstrates the performance of our key modules. (2) To evaluate the contribution of each module, we designed ablation studies. (3) Due to the pipeline is designed to be applied to the real world, we also validate our pipeline’s robustness under different challenging environments.

### 4.1. Experiment Setup

#### 4.1.1. Data Preparation

Compared with the widely used datasets in deep learning such as PASCAL VOC [43], COCO [44] and ImageNet [45], to the best of our knowledge, there is no public watermeter dataset with reading annotations. To foster the training for watermeter reading, we collected watermeter images from real life and web crawlers. We hired people to the houses where people actually live and collect watermeter original data by manually taking pictures. After that, we hired people with labeling experience to label the training data through VIA [46] (a simple and powerful manual image annotation tool). The watermeter images in the resulting dataset contain a variety of angles, colors, lighting, resolutions, and background scenes, etc. We randomly divide the collected data set into training data and test data at a ratio of 95% and 5%.

**Figure 8:**
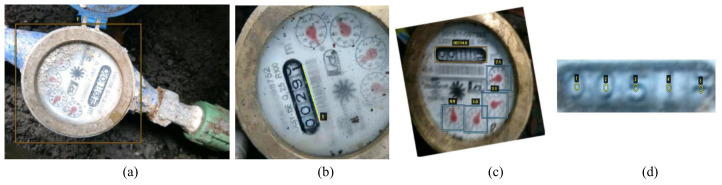
Examples of data annotations required to train different models. (**a**) annotates the position of the water meter, (**b**) annotates the direction of watermeter, (**c**) annotates the position and value of the inner components, and (**d**) annotates the digit box’s center points.

As shown in Figure 3, six models need to be trained in our system, and they are executed sequentially. The latter model depends on the previously trained model. Therefore, we annotate our training data progressively. As shown in Figure 8a, we first annotate the position of the watermeter at the original collected images. Model $M_{1}$ is trained on these annotated images, and the trained model is used to detect watermeter on all original images. Detected watermeters are cropped out, and we can obtain images like Figure 8b. Secondly, we annotate the line segment with direction at cropped watermeter. Then, we calculate the angle based on the annotated line segment and then use the sin and cos values of the angle to supervise the training of $M_{2}$. Trained $M_{2}$ is then used to correct the orientation of all training data. Thirdly, we annotate the bounding boxes and actual values of digits and pointers, as shown in Figure 8c. With annotated position supervision, we train another detection model $M_{4}$. Trained $M_{4}$ is then used to crop out digits and pointers. Finally, $M_{6}$ is trained with cropped pointer image and annotated value. $M_{5}$ is trained with cropped digits image and annotated digit value. Furthermore, five-digit center points in the digit box, as shown in Figure 8d, are annotated to guide the center localization.

#### 4.1.2. Implementation Details

The backbone of orientation alignment $M_{2}$, spatial layout guidance $M_{4}$ and pointer regression $M_{6}$ are modified versions of the ResNet-50 [47] excluding the average pooling layer. We initialized all models with pretrained weights (YOLO3 pretrained on the COCO [44] dataset for object detection and ResNet pretrained on the ImageNet [45] dataset for image recognition). For each model, we split the training process into two stages. We update only the last layer of the model for the first stage and then update all layers together for the second stage. We use the Adam [48] optimizer and a learning rate of $10^{-4}$ are employed for optimization. Input image sizes for the six models are (416×416), (416×416), (224×224), (224×224), (32×32) and (224×224).

#### 4.1.3. Evaluation Metrics

We used three evaluation metrics in the experiments: angle error, digit error and pointer error. The output of digit is an integer, so we can judge it directly. The output of pointer is a decimal, and it is judged by whether its value is within ±0.5 of the ground truth. The calculation formulas for other evaluation metrics are as follows:

(4) $$Angle_{error}=∥{Angle}_{truth}-{\hat{Angle}}_{pred}∥,$$

(5) $$Digit_{error}=\frac{{Digits}_{wrong}}{{Digits}_{all}},$$

(6) $$Pointer_{error}=\frac{{Pointer}_{wrong}}{{Pointer}_{all}},$$

where ${Angle}_{truth}$ denotes the groud truth rotation angle of the watermeter, ${\hat{Angle}}_{pred}$ denotes the predicted rotation angle of the watermeter. ${Digits}_{wrong}$ denotes the number of incorrectly predicted digits, ${Digits}_{all}$ denotes the total number of digits. And ${Pointer}_{wrong}$ denotes the number of incorrectly predicted pointers, ${Pointer}_{all}$ denotes the total number of pointers.

### 4.2. Performance Evaluation for Key Modules

#### 4.2.1. Orientation Alignment

**Figure 9:**
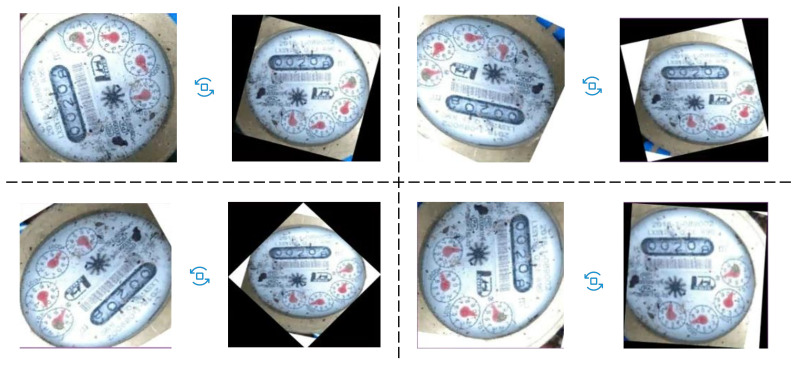
Qualitative results of orientation alignment. Regardless of how much the image angle shifted, the orientation alignment module adjusts it correctly.

We eliminate the ambiguity of the angle periodicity by predicting the sin and cos values of the angle. Then we calculate the angle according to these values. To test the performance of our orientation alignment model $M_{2}$ in different rotation intervals, we first correct the original data according to the annotation pair and then rotate it randomly. The rotation angle meets the uniform distribution of different intervals. The results of each interval are given in Table 1 and the qualitative results are shown in Figure 9. The average error between the actual angle and recognition angle is less than 1 degree. The experimental result illustrates that the method finely amends the error caused by the slanted image.

In addition, the orientation alignment module also plays a key role in guiding the recognition rate and accuracy of the digit box. For this purpose, we conducted tests on the test dataset (see the results in Table 2).

#### 4.2.2. Spatial Layout Guidance for Digit Localization

**Figure 10:**
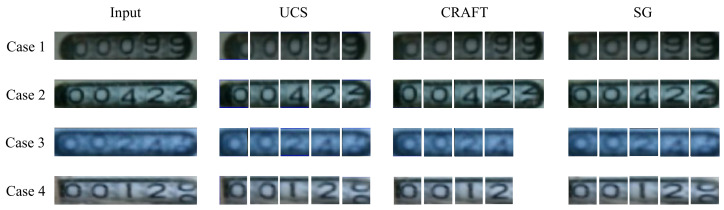
Comparison of segmentation methods. UCS is the uniform character segmenting, CRAFT is the OCR text detector, and SG is the spatial guidance method we proposed.

We determine that $M_{3}$ is able to identify the position of the digit box with high precision. Therefore, we directly utilize the uniform character segmentation method to divide each digit. After testing, however, we found that if the position identified by $M_{3}$ is not accurate, the segmentation result will be poor (Figure 10 Case 1 and Case 2 in the second column). Therefore, to decouple the digital positioning from the previous step, we utilize the OCR text detector CRAFT [41] to detect each digit. CRAFT is not accurate enough, however, when identifying a rolling digit (Figure 10 Case 3 and Case 4 in the third column). We thus propose the use of spatial guidance before solving this problem. The visual comparison of these methods are shown in Figure 10.

Besides, to verify the digit localization module’s stability, we expanded the test dataset four times by data augmentation methods (e.g., random rotation, scaling, and color transformation) for quantitative experiments. After data augmentation, the difficulty increases, which will inevitably lead to an increase in the error rate. However, the error rate of SG grew slightly, while others’ error rates grew significantly. The results are shown in Table 3. We use the error growth rate (error growth rate is the grew digit error ratio the original digit error) as an evaluation metric to make a more intuitive comparison. The tilt of the angle increases the error rate of UCS (digit error increased by 2.32%, with an error growth rate of 27%); the recognition rate of CRAFT is worse due to the color transformation and thus increases the error rate of readings (digit error increased by 1.04%, with an error growth rate of 18%); while the error rate of SG increase in a small range (digit error increased by 0.25%, with an error growth rate of 6%), which means SG stability in to various environments.

### 4.3. Ablation Studies

To understand the role of the orientation alignment and the proposed spatial layout guidance, and to test the effectiveness of each proposed module, we performed ablation studies on our test dataset.

#### 4.3.1. Effectiveness of Orientation Alignment

We first explore the effectiveness of the proposed orientation alignment module. To validate the effectiveness of orientation alignment, we removed this module and analyzed the impact on performance. Table 3 shows the effect of orientation alignment on automatic reading. After adding the orientation alignment module, the reading accuracy is significantly improved. This demonstrates that the orientation alignment module is critical for the automatic reading.

#### 4.3.2. Effectiveness of Spatial Guidance

In Section 4.2, we proved that the spatial guidance method could cope with inaccurate $M_{3}$ results and rolling digits. We further compare the influence of the presence or absence of spatial guidance on the reading results. It was evident that each component significantly improves our results, and the spatial guidance method plays an essential role in automatic reading.

**Figure 11:**
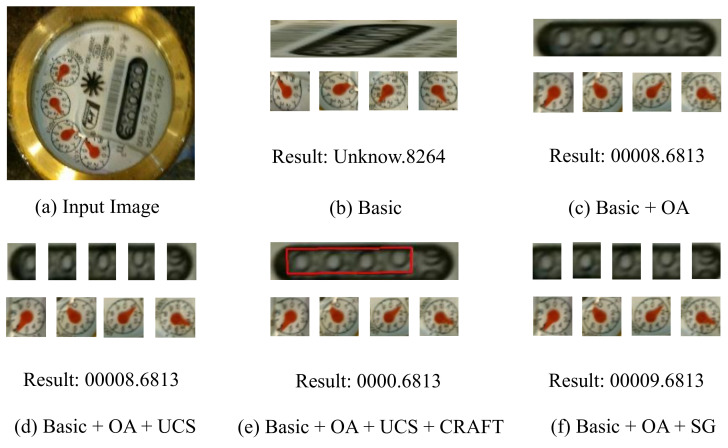
Visual comparison of different methods. “Basic+” represents that we add different components to the baseline to the baseline network to read the watermeter automatically.

A visual comparison is provided in Figure 11. Basic reading failed because the reading is conducted at the wrong orientation (Figure 11b). UCS may have segmented incorrectly, resulting in erroneous readings (Figure 11d). Meanwhile, CRAFT may have made incomplete detections, resulting in incorrect readings (Figure 11e). OA could provide the correct angle for the reading (Figure 11c) and SG accurately segment the digits in the digit box (Figure 11f); notably, their aggregation could generate high-quality reading results.

### 4.4. System Performance

This section provides the results of the system’s performance test and briefly describes the deployment of the system.

#### 4.4.1. Robustness to Challenging Environments

**Figure 12:**
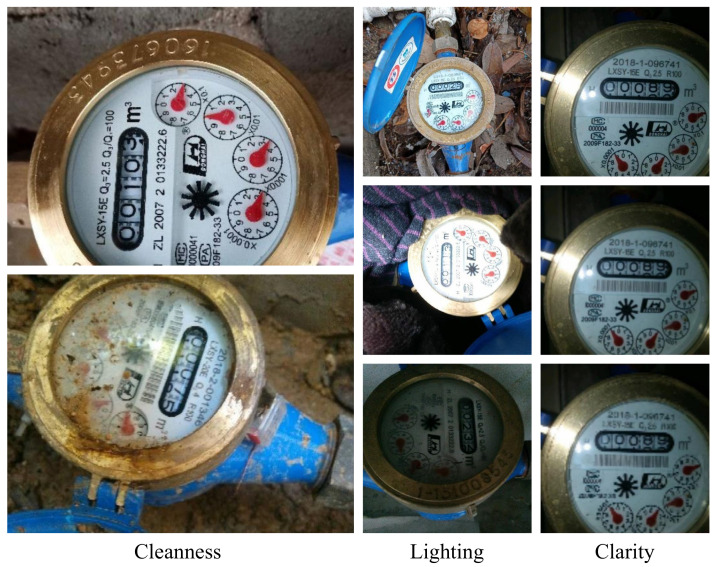
The test dataset images with different cleanliness, lighting and clarity.

To further validate our pipeline’s robustness, we subdivided the test dataset into three major categories based on cleanness, lighting conditions, and image clarity (see Figure 12). The cleanliness is subdivided into normal and dirty; the lighting environment is subdivided into normal, bright and dark. The image clarity is subdivided into the original sharpness, and the sharpness is reduced twice and three times. There are a total of seven sub-categories, each with 100 pictures. We use the digit error and the pointer error as evaluation metrics, the experimental results are shown in Table 4.

**Figure 13:**
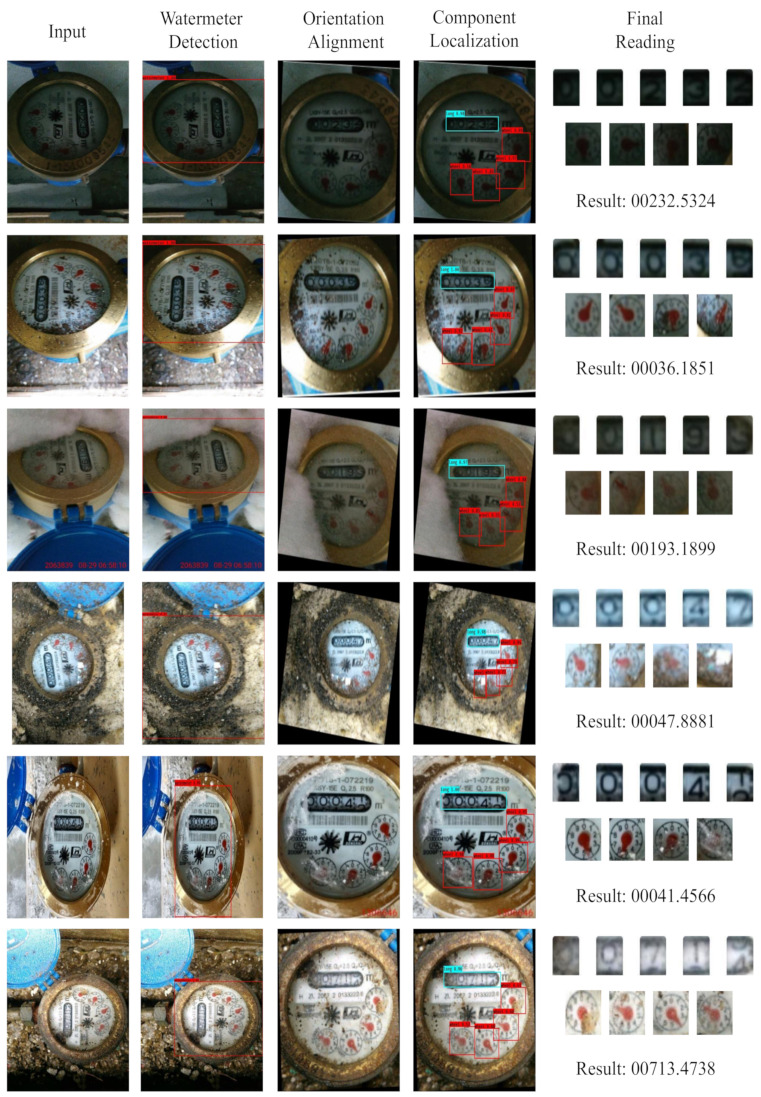
Performance test of the complete system: the first column is the input, the second to fourth columns are the intermediate results, and the last column is the final reading result.

Experimental results show that our proposed pipeline has the lowest error rate in various environments. Figure 13 shows some of these results: these watermeters had different angles and perspectives; some watermeters were located in dark or light-reflective environments and some watermeters were blurry and covered with dust. Under these various challenging environments, our method achieved satisfactory results.

#### 4.4.2. System Deployment

**Figure 14:**
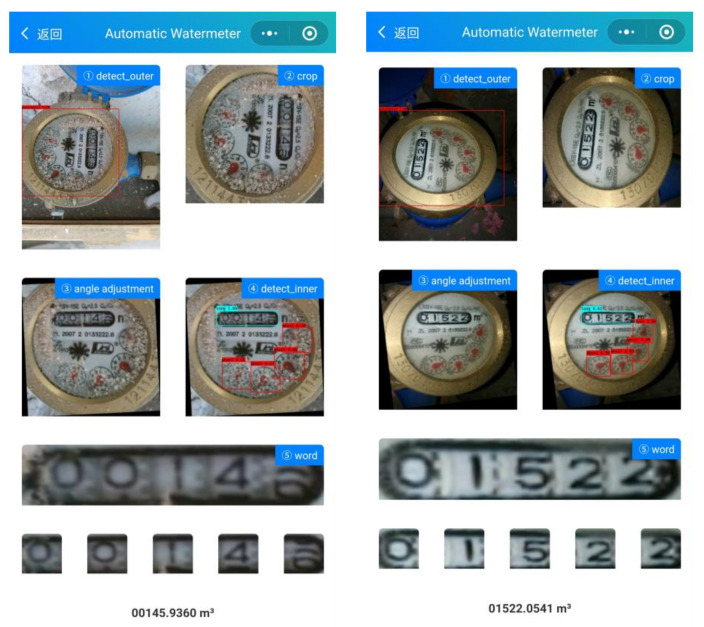
The user interface of the system. Just click to upload the image to complete the meter reading.

Based on the proposed method, we developed and deployed an online system for automatic meterwater reading. The system is constructed with Flask, a Python micro-framework to provide API services. All the models are preloaded and run on a PC with an i7 CPU and a single Nvidia GTX 1080 graphics card. The average time to infer an image is less than 300 ms. The visual interface of the system is shown in Figure 14.

#### 4.4.3. Failure Case

**Figure 15:**
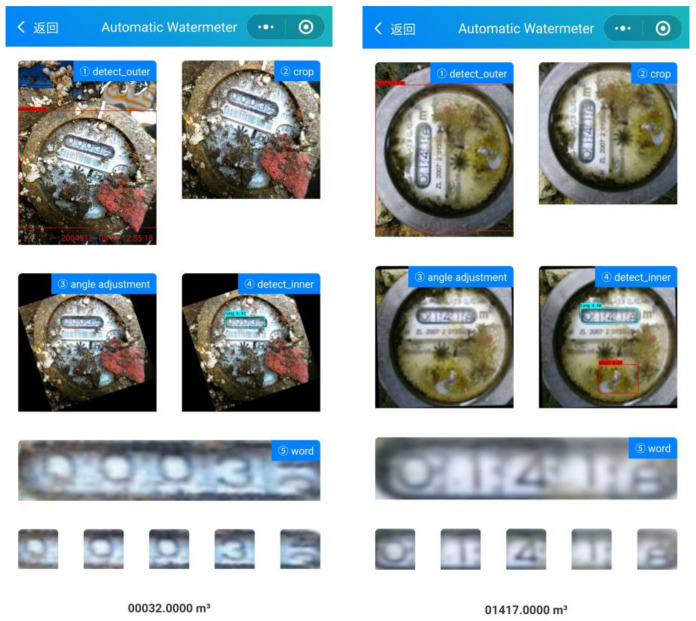
Failure cases. The pointer is partially obscured and cannot be recognized, and therefore cannot be read.

Our system is able to deal with various challenging environments, including darkness, blur, glare, dirt, and distant capture, etc. The network, however, could not handle some situations. As shown in Figure 15, when the input image is covered by foreign objects, our system fails to make a reading.

## 5. Conclusions

In this paper, we propose a fully automatic system for watermeter reading. It is based on end-to-end CNN, including watermeter detection and rotation corrected component localization, regression-based digit reading with spatial layout guidance, and pointer reading. We construct a new watermeter dataset containing images obtained in various challenging environments for system training and testing. Extensive experiments prove that the orientation alignment module can effectively improve the digit box and pointer detection accuracy; the spatial guidance module can effectively improve the digit box’s reading accuracy. Besides, we conducted quantitative experiments to verify the orientation alignment module’s efficiency and the spatial guidance module. Moreover, we designed ablation experiments to verify each module’s effectiveness in the whole pipeline. To sum up, our method can successfully automatically read water meters under challenging environments with high accuracy, which meets the practical application’s requirements.

In future work, we will further improve the accuracy of automatic readings in the actual application process to reduce failure cases. For foreign object coverage, the system will notify the staff to be manual clean; for others, the system will collect these images as part of our dataset. Expanding the real-world environment dataset, we will continue to finetune our model on the expanded data to improve our pipeline’s robustness and reduce the error rate.

## Figures and Tables

**Table 1:** The quantitative results for the orientation alignment module. The evaluation metric is the angle error.

Angle Distribution	Max Ang. Err.	Min Ang. Err.	Average Ang. Err.
*U*(−10°, 10°)	3.488°	0.001°	0.688°
*U*(−20°, 20°)	3.736°	0.001°	0.713°
*U*(−30°, 30°)	4.690°	0.004°	0.700°
*U*(−40°, 40°)	4.363°	0.003°	0.822°
*U*(−50°, 50°)	3.131°	0.001°	0.735°
*U*(−60°, 60°)	4.791°	0.001°	0.753°
*U*(−70°, 70°)	3.982°	0.001°	0.750°
*U*(−80°, 80°)	3.585°	0.003°	0.726°
*U*(−90°, 90°)	4.026°	0.001°	0.709°

**Table 2:** Effectiveness of orientation alignment on the recognition of the digital box. Average IOU and AP are used as evaluation metrics for detecting digit.

Orientation Alignment	Average IOU	AP@0.5
	0.51	42.11
✓	0.92	98.92

**Table 3:** Ablation study on the test dataset. “*” represents the test dataset with data augmentation. “Basic” means our baseline network. OA denotes orientation alignment, UCS denotes uniform character segmentation, and SG denotes spatial guidance. The evaluation metrics are the digit error and the error growth rate.

Approach	Basic	OA	UCS	CRAFT	SG	Digit Err.
Basic	✓					24.32%
Basic + OA	✓	✓				13.20%
Basic + OA + UCS	✓	✓	✓			8.52%
Basic + OA + UCS + CRAFT	✓	✓	✓	✓		5.72%
Basic + OA + SG	✓	✓			✓	3.79%
* Basic	✓					35.86% (+47%)
* Basic + OA	✓	✓				17.76% (+34%)
* Basic + OA + UCS	✓	✓	✓			10.84 (+27%)
* Basic + OA + UCS + CRAFT	✓	✓	✓	✓		6.76% (+18%)
* Basic + OA + SG	✓	✓			✓	4.04% (+6%)

**Table 4:** The Quantitative experiments for the pipeline’s robustness. “Down” means zoom out and then zoom in to the original image, and the number after it is multiple. The evaluation metrics are the digit error and the pointer error.

		Digit Err.		Pointer Err.	
		Base	Base + OA + SG	Base	Base + OA + SG
**Cleanness**	**Normal**	15.0%	3.4%	1.0%	1.0%
	**Dirty**	19.2%	3.6%	7.0%	3.0%
**Lighting**	**Normal**	11.4%	3.4%	2.0%	1.0%
	**Bright**	13.9%	3.6%	4.0%	2.0%
	**Dark**	14.2%	3.8%	4.0%	2.0%
**Clarity**	**Normal**	13.8%	2.0%	1.0%	0.0%
	**Down × 2**	14.6%	2.2%	2.0%	2.0%
	**Down × 3**	16.4%	4.0%	7.0%	3.0%

## Data Availability

The data presented in this study are available on request from the corresponding author.
